# Early identification of disease progression in ALK-rearranged lung cancer using circulating tumor DNA analysis

**DOI:** 10.1038/s41698-021-00239-3

**Published:** 2021-12-07

**Authors:** Arlou Kristina Angeles, Petros Christopoulos, Zhao Yuan, Simone Bauer, Florian Janke, Simon John Ogrodnik, Martin Reck, Matthias Schlesner, Michael Meister, Marc A. Schneider, Steffen Dietz, Albrecht Stenzinger, Michael Thomas, Holger Sültmann

**Affiliations:** 1 Division of Cancer Genome Research, German Cancer Research Center (DKFZ), German Cancer Consortium (DKTK), and National Center for Tumor Diseases (NCT), Heidelberg, Germany; 2 Translational Lung Research Center Heidelberg, German Center for Lung Research (DZL), Heidelberg, Germany; 3 Department of Oncology, Thoraxklinik and National Center for Tumor Disease (NCT) at Heidelberg University Hospital, Heidelberg, Germany; 4 Bioinformatics and Omics Data Analytics, German Cancer Research Center (DKFZ), Heidelberg, Germany; 5 Medical Faculty, Heidelberg University, Heidelberg, Germany; 6 Lung Clinic Grosshansdorf, Airway Research Center North, German Center for Lung Research, Grosshansdorf, Germany; 7 Translational Research Unit, Thoraxklinik at Heidelberg University Hospital, Heidelberg, Germany; 8 Institute of Pathology, Heidelberg University, Heidelberg, Germany; 9 German Cancer Consortium (DKTK), Heidelberg, Germany; 10 Present Address: Biomedical Informatics, Data Mining and Data Analytics, Faculty for Applied Informatics, Augsburg University, Augsburg, Germany; 11 Present Address: AstraZeneca GmbH, Wedel, Germany

**Keywords:** Non-small-cell lung cancer, Cancer genomics

## Abstract

Targeted kinase inhibitors improve the prognosis of lung cancer patients with *ALK* alterations (ALK+). However, due to the emergence of acquired resistance and varied clinical trajectories, early detection of disease progression is warranted to guide patient management and therapy decisions. We utilized 343 longitudinal plasma DNA samples from 43 ALK+ NSCLC patients receiving ALK-directed therapies to determine molecular progression based on matched panel-based targeted next-generation sequencing (tNGS), and shallow whole-genome sequencing (sWGS). *ALK-*related alterations were detected in 22 out of 43 (51%) patients. Among 343 longitudinal plasma samples analyzed, 174 (51%) were ctDNA-positive. *ALK* variant and fusion kinetics generally reflected the disease course. Evidence for early molecular progression was observed in 19 patients (44%). Detection of ctDNA at therapy baseline indicated shorter times to progression compared to cases without mutations at baseline. In patients who succumbed to the disease, ctDNA levels were highly elevated towards the end of life. Our results demonstrate the potential utility of these NGS assays in the clinical management of ALK+ NSCLC.

## Introduction

About 3–7% of non-small-cell lung cancer (NSCLC) belong to a molecular subgroup defined by the presence of *ALK* (anaplastic lymphoma kinase) rearrangements^1^. Since its discovery, *ALK*-rearranged (ALK+) NSCLC has been a model disease for targeted therapies using tyrosine kinase inhibitors (TKIs) that potently attenuate the function of ALK^2–5^. While initial responses to selective TKIs are durable, ALK+ tumors eventually and inevitably develop resistance to targeted therapy. For example, the first FDA/EMA-approved ALK inhibitor crizotinib elicited improved objective response rates and progression-free survival (PFS) in randomized phase III trials compared to chemotherapy, but disease relapse was nonetheless observed within 1 year of treatment^6^. Consequently, significant insights into the molecular underpinnings of TKI therapy resistance have been gained. At the same time, increasingly potent and selective next-generation ALK TKIs have also been developed and approved for clinical use^7–10^. Although sequential therapy using next-generation ALK inhibitors improves PFS and mitigates brain metastases^11^, ALK+ tumors continue to adapt and develop alternative resistance mechanisms against these drugs.

The dynamic adaptability of ALK+ tumors against ALK inhibitors requires a personalized approach to disease monitoring for accurate and timely clinical patient management. Next-generation sequencing (NGS)-based analysis of tissue DNA is an established and a highly robust method for baseline genotypic evaluations^12,13^. However, while longitudinal tissue biopsies could identify emerging resistance mutations, this strategy is limited by a number of factors: (a) single-site tumor biopsies would most likely fail to capture the full spectrum of genomic alterations of a molecularly heterogeneous tumor^14^; (b) multiple tumor re-biopsies pose procedural risks, and sometimes are not feasible^15^; and (c) tissue sample preparation such as formalin fixation could lead to false-positive results in molecular assays due to high levels of base transitions^15,16^. Therefore, liquid biopsy technologies are currently emerging as minimally invasive and easily accessible alternatives to tissue biopsies. Peripheral blood plasma naturally harbors molecular components that can be analyzed and attributed to certain pathologies. Among these, the circulating tumor DNA (ctDNA) compartment provides highly sensitive and insightful genetic information for evaluating tumor heterogeneity and clonal evolution. In addition to being a surrogate for localized tissue biopsy, ctDNA has the potential to capture the complete genomic profile of the primary tumor and metastases at expansive time points without spatial bias. Detection of such exhaustive genetic tumor profiles is particularly relevant for disease monitoring of patients undergoing treatment, where tumor subclones could emerge due to selective pressures introduced by various therapeutic challenges.

Previously, overall ctDNA levels have been utilized as independent biomarkers of disease progression or therapy response in multiple cancer entities including metastatic breast cancer^17^, melanoma^18^, metastatic gastrointestinal cancer^19^, metastatic bladder cancer^20^, and lung cancer^21^. ctDNA detection has also been explored for prediction of disease recurrence and minimal residual disease^22–24^. In ALK+ NSCLC, liquid biopsies have successfully been utilized for *ALK* resistance mutation profiling, presenting important implications for therapy decisions^21,25,26^.

We first reported the potential clinical utility of a combination of cfDNA assays for longitudinal monitoring of ALK+ NSCLC under TKI therapy^27^. We performed matched capture-based high coverage targeted NGS (tNGS) using a commercially available panel comprised of genes optimized for longitudinal tumor burden monitoring in lung and colorectal cancers. Simultaneously, we applied the trimmed median absolute deviation from the copy number neutral state (t-MAD) score using shallow whole-genome sequencing (sWGS) to quantify global copy number changes derived from ctDNA^28^. The information derived from these ctDNA assays revealed the complex mutational kinetics of ALK+ tumors across therapy lines, as well as the increasing genomic instability of the tumor after sequential TKI treatments. Here, we extend the number of longitudinal plasma samples from our initial ALK+ NSCLC cohort and for the first time, using tNGS and sWGS data, identify threshold values for changes in variant allele frequencies (VAFs) and t-MAD scores that potentially indicate disease progression earlier than current conventional modalities. With additional sampling points, we show that a combined NGS assay approach can reveal molecular progression that precedes radiographic assessment, particularly in patients with detectable ctDNA. Furthermore, our study cohort demonstrates the potential of targeted sequencing in baseline risk stratification of patients, and corroborates previous data about the negative prognostic impact of *TP53* alterations detected during therapy.

## Results

### Patient characteristics

**Fig. 1:**
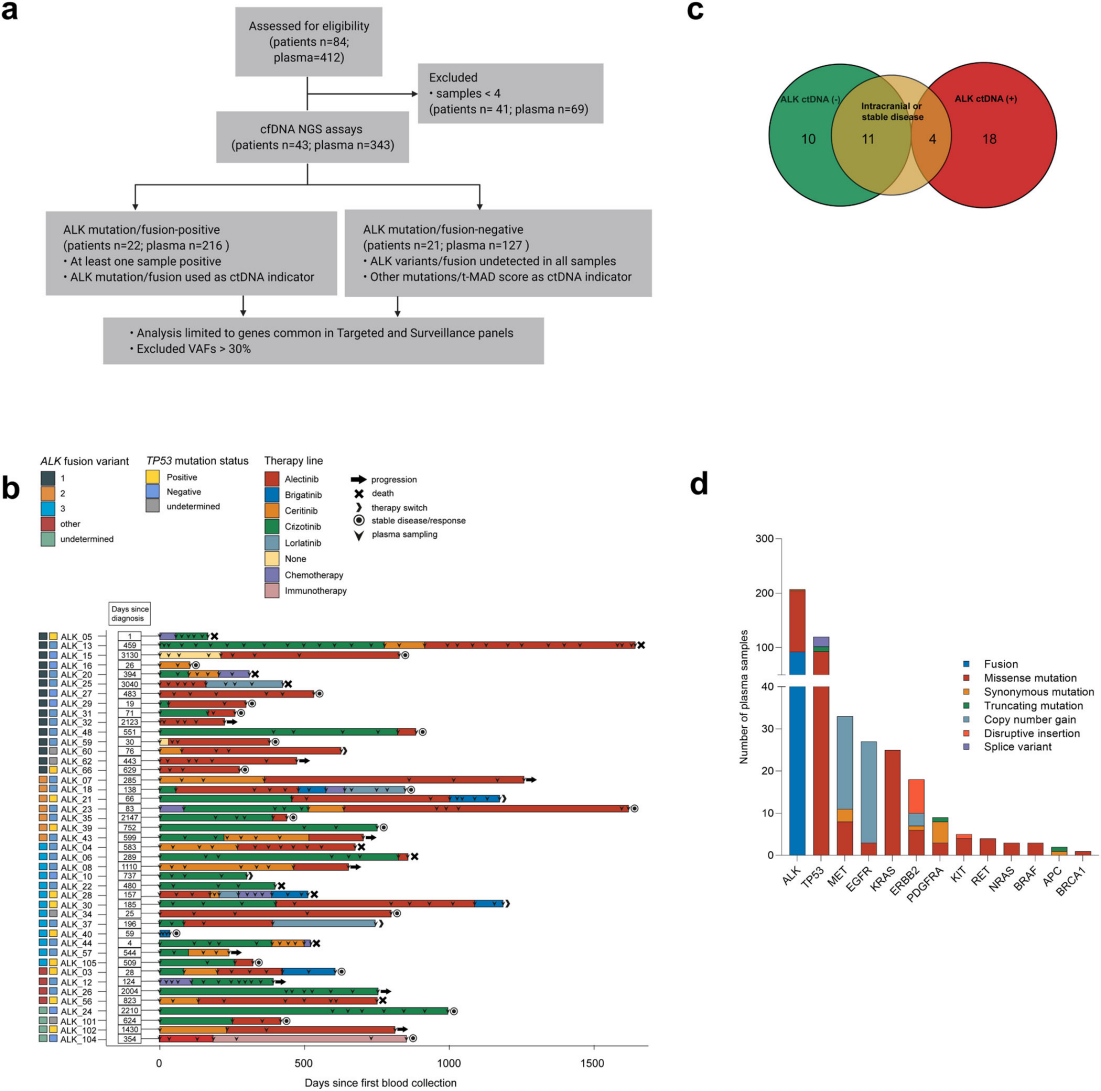
Overview of the ALK+ NSCLC cohort. **a** Untargeted sWGS and targeted NGS assays were used to assess ctDNA in 43 ALK+ NSCLC patients corresponding to 343 longitudinal plasma samples. Targeted NGS analysis only included genes common to both the Avenio Targeted and Surveillance panels. In total, 51% (22 of 43) of patients had an *ALK* alteration in at least one liquid biopsy. **b** Timeline of plasma collection and therapy administration in the patient cohort. *ALK* fusion variants and *TP53* status from tissue analysis at diagnosis are indicated by colored boxes. **c** Distribution of patients based on detected *ALK* alterations—including mutations and rearrangements. The group without detectable *ALK* alterations (ALK−) is enriched for patients with intracranial progression or stable disease. **d** Molecular alterations identified in 174 ctDNA (+) plasma samples based on tNGS.

In total, 43 metastatic ALK+ patients with corresponding 343 longitudinal plasma samples were considered for NGS analysis (Fig. 1a, see Methods). The median age of the study population was 57 years (range 39–80), with almost equal distribution of males and females (53% males). Seventy eight percent of patients were never/light-smokers (<10 pack-years, Table 1). Based on tissue biopsies, *EML4-ALK* variant 1 (V1, E13:A20) was identified in 35% (15/43), V2 (E20:A20) in 16% (7/43), V3 (E6:A20) in 30% (13/43), and other variants in 9% (4/43) of tumors. Other cases of *ALK* fusion were detected with tissue RNA-NGS at initial diagnosis. *TP53* mutation were detectable in 11/39 (28%) patients with available tissue DNA at diagnosis. The therapy regimens that patients underwent at each sampling time point are shown in Fig. 1b. The first-generation TKI crizotinib was administered to 25/43 patients. Second-generation TKIs (ceritinib, alectinib, brigatinib) were administered to 36/43 patients, and third-generation TKI lorlatinib was given to 4/43 patients.

**Table 1:** Patient characteristics.

ALK+ NSCLC patients analyzed in this study (*n* = 43)		
Age, median (39–80)		57 (10)
Sex, % male		53%
Smoking status (% never smokers)^a^		78%
ECOG PS (%) at baseline		
0		26
1		14
2		1
No data		2
Histology^b^		
adenocarcinoma		42/43
*ALK* fusion variant^c^		
* EML4-ALK* V3		13
* EML4-ALK* V1/V2		22
Other		4
No data		4
*TP53* status at baseline, mutated^d^		11/39
ALK TKI, patient number		
Crizotinib		25
Ceritinib/alectinib/brigatinib		36
Lorlatinib		4
Chemotherapy		7
Follow-up in months (median, [Q3-Q1])		37 (52–27)
Number of samples analyzed per patient (mean [range])		8 (4–31)
Percentage of cases with treatment-naive samples		21%
Number of samples at disease progression per patient, mean		2.8
Number of TKI lines covered with liquid biopsy per patient, mean		1.9

*SD* standard deviation, *EML4-ALK* echinoderm microtubule-associated protein-like 4 (*EML4*) and anaplastic lymphoma kinase (*ALK*) fusion, *PS* performance status, *TKI* tyrosine kinase inhibitor.

^a^Data available for 41/43 cases.

^b^One patient had an ALK+ large-cell neuroendocrine lung carcinoma responsive to ALK inhibitors.

^c^Data available for 39/43 cases; one case with E18A20, one with E9A20, one with K9A20 (*KCL1*), and one with K24A20 (*KIF5B*).

^d^Data available for 39 cases by NGS of tissue biopsies at diagnosis of stage IV disease.

### Genomic alteration landscape of ALK+NSCLC ctDNA

Among the patients under study, 22 (51%) had detectable *ALK* alterations (mutation or rearrangement) in at least one plasma sample based on tNGS profiling (Fig. 1a and Supplementary Table 1). A total of 36 *ALK* mutational events were detected, 34 of which were known or probable resistance mutations, while two were silent mutations. Of the remaining 21 patients, 11 (52%) had either intracranial progression (*n* = 6) or stable disease at all sampling points (*n* = 5), potentially explaining the low amount of ctDNA shedding and unmeasurable *ALK* alterations in plasma DNA (Fig. 1c). ctDNA was detected in a total of 174/343 (51%) plasma samples. Most of these were missense, synonymous, and truncating mutations, disruptive inframe insertions, as well as fusions and copy number changes in *ALK* and *TP53* with 56% and 52% detection rate in ctDNA (+) plasma samples, respectively. Other genes prominently altered were *MET*, *EGFR*, *KRAS*, *ERBB2*, *PDGFRA*, *KIT*, *RET*, *NRAS*, *BRAF*, *APC*, and *BRCA1* (Fig. 1d). Copy number gains were also detected in *MET*, *EGFR*, and *ERBB2*.

### Dynamics of *ALK* rearrangement and resistance mutations during TKI therapy

We interrogated the concordance between *ALK* mutation or fusion abundance in *ALK* ctDNA+ (*n* = 22) patients and disease status. Emergence of at least one *ALK* mutation coincided with progressive disease in eight of these patients (Supplementary Table 2), while seven patients (ALK_06, 13, 18, 20, 25, 44, 62) showed increasing *EML4-ALK* fusion abundance upon progression on independent therapy lines (Supplementary Fig. 1). Five patients developed multiple secondary mutations (ALK_12, 13, 28, 44, 62), which included known TKI therapy resistance variants I1171N, I1171T, F1174L, F1174V, F1174C, L1196M, G1202R, and G1269A^29^. The median VAF of these *ALK* mutations was 0.18%. During the follow-up period, four patients (ALK_03, 13, 18, and 28) underwent subsequent treatment with a TKI that targeted the *ALK* mutation (Supplementary Fig. 1). In all cases, a reduction of the VAF of the mutation was observed after the change of therapy until the onset of further disease progression.

### Prognostic utility of ctDNA

**Fig. 2:**
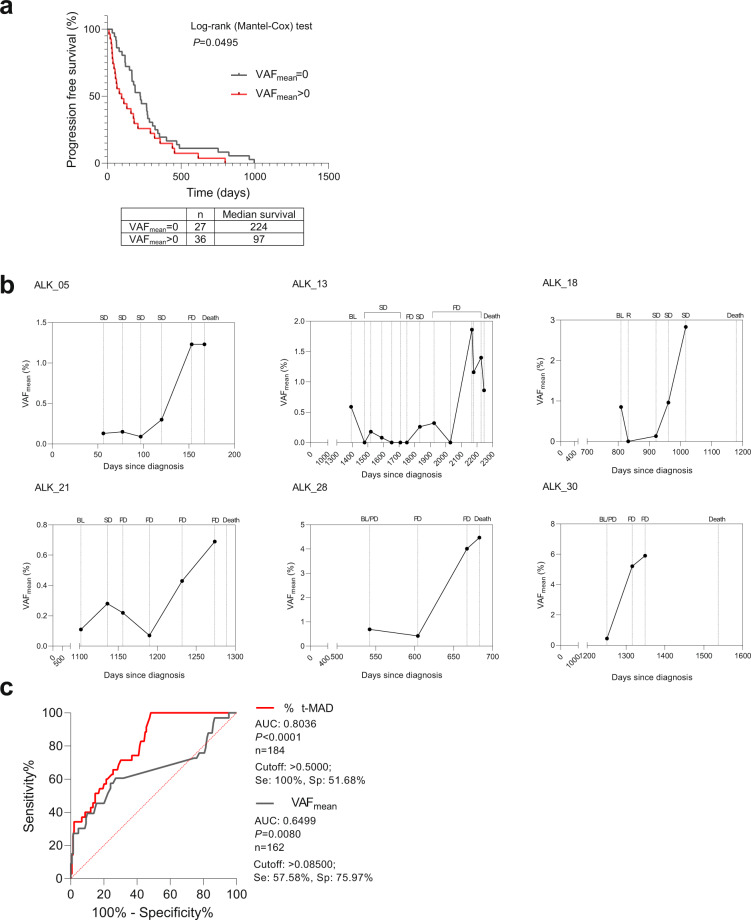
Prognostic utility of ctDNA NGS assays. **a** Mean variant allele frequency at baseline indicates therapy durability. Progression-free survival plot showing significantly longer therapy durability in instances when ctDNA is undetected (VAF_mean_ = 0) compared to detection (VAF_mean_ > 0) at treatment initiation. **b** VAF kinetics in representative patients showing elevated VAF_mean_ towards end of life. SD stable disease, PD progressive disease, BL therapy baseline, R response. **c** Comparison of receiver operating characteristic curves (ROC) using ΔVAF_mean_ (gray) or %Δt-MAD (red) in discriminating progressive and non-progressive disease states.

We analyzed the relevance of detecting variants at treatment initiation in relation to subsequent therapy durability, indicated by the time to progression from treatment baseline. We determined that upon independent evaluation of each therapy instance, detection of VAF (i.e., VAF_mean_ > 0) at therapy baseline reflected significantly shorter time to progression compared to cases without detectable variants (median: 97 vs. 224 days) (*P* < 0.0495; Fig. 2a). Additionally, we observed elevated ctDNA levels as measured by VAF_mean_ towards the end of life in 14 out of 16 patients who eventually succumbed to the illness. In six representative patients, the measured ctDNA levels were among the highest in samples prior to death (Fig. 2b).

### Evaluating the potential utility of changes in genome-wide copy number and VAF_mean_ as indicators of disease progression

We asked whether the t-MAD score, as a measure of global copy number changes derived from ctDNA, could be an indicator of clinical progression. Since the range of absolute t-MAD scores for each patient varied due to the heterogeneity of clinical status and applied therapy lines, we utilized a relative measurement of t-MAD based on percentage of change (%Δt-MAD) to determine whether this score of genome-wide copy number aberration could indicate disease progression. We found that the relative change in t-MAD score at successive sampling points was associated with extracranial progressive disease coincident with therapy change (AUC: 0.8036 [0.7333–0.8740], *P* < 0.0001, Fig. 2c). The cutoff with maximum sensitivity (100%) and specificity (51.68%) was a t-MAD score increase of 0.5%.

**Fig. 3:**
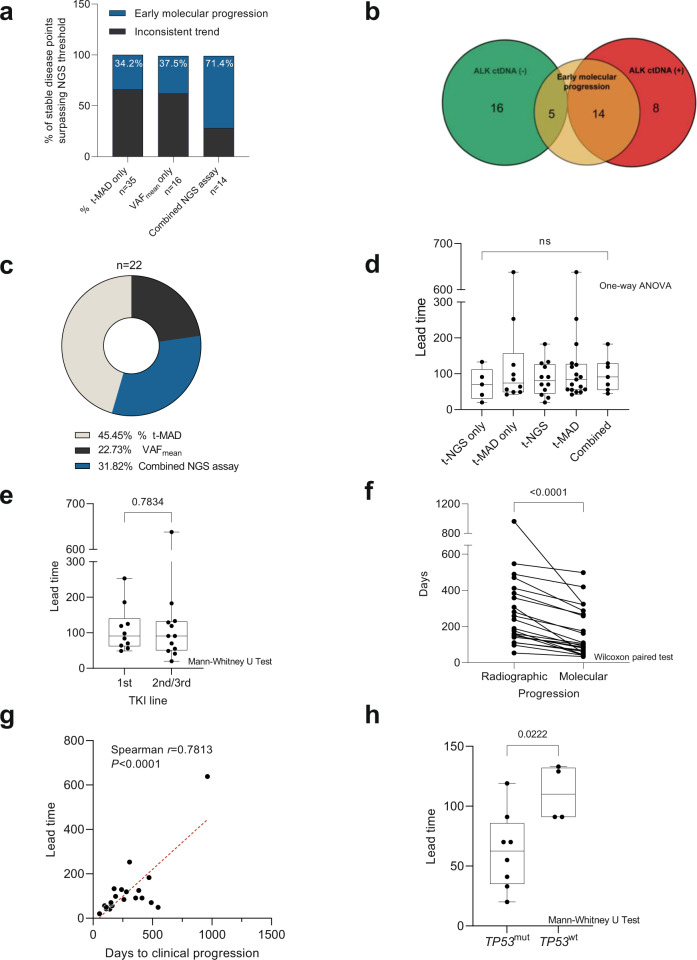
Evaluation of the utility of ΔVAF_mean_ and %Δt-MAD in identifying early molecular progression. **a** Number of stable disease points that surpassed the numerical cutoffs per individual NGS assay (%Δt-MAD or ΔVAF_mean_) and in combination. Gray stacks indicate the percentage of points with inconclusive NGS assay values leading to clinical progression. Blue stacks represent points that showed sustained %Δt-MAD and/or ΔVAF_mean_ increase leading to clinical progression, indicating early molecular progression. **b** Patients identified with early molecular progression were enriched in the ALK ctDNA (+) group. **c** Breakdown of 22 patients with early molecular progression based on NGS assays. **d** Comparison of called lead times based on NGS assays. **e** Comparison of called lead times based on therapy lines. **f** Comparison of cases with called lead time showing significant difference in days to radiographic progression versus molecular progression. **g** Length of lead time per therapy line was significantly correlated with duration of progression-free response to the respective treatment. **h** Lead times in cases with wild-type *TP53* (*TP53*^wt^) were significantly longer compared to cases with mutated *TP53* (*TP53*^wt^) detected by tNGS. The box plot show the the median, upper quartile, and lower quartile values. The whiskers indicate minimum and maximum values.

Similarly, ctDNA levels based on tNGS were applied to assess and predict therapy response^17,30–33^. Here, we used the difference between the detectable VAF_mean_ (ΔVAF_mean_) of consecutive sampling points to quantify the change in ctDNA abundance^31^. The ΔVAF_mean_ also correlated significantly with clinical status (i.e., extracranial progressive disease coincident with therapy change), but with a lower AUC than %Δt-MAD (AUC: 0.6499 [0.5277–0.7720], *P* = 0.0080, Fig. 3b). The optimal ΔVAF_mean_ cutoff was 0.0850, which showed a sensitivity of 57.58% and specificity of 75.97% (Fig. 2c).

### Molecular progression precedes clinical progression in a subset of ctDNA (+) patients

Next, we investigated whether ctDNA detection, as indicated by molecular progression, could predate and thus predict eventual clinical progression. Here, we used the ΔVAF_mean_ and %Δt-MAD cutoff values defined from the ROC curve analysis of progressive disease points with therapy change as described above (i.e., ΔVAF_mean_ = 0.0850, %Δt-MAD = 0.500%). Due to the heterogeneous therapy regimens across patients, we evaluated the time difference in days between early molecular and clinical progression (i.e., lead times) independently for each therapy line per patient. To identify early molecular progression, we examined sampling time points under treatment with stable disease according to RECIST v1.1. We identified 30 and 49 instances of such molecular progression using ΔVAF_mean_ and %Δt-MAD, respectively, with 14 instances meeting both thresholds (Fig. 3a). Subsequently, we assigned a lead time if the initial increase in ctDNA was sustained at subsequent sampling points and eventually led to a progressive disease state. This second criterion validated 6 out of the 16 (37.5%) points based on ΔVAF_mean_ alone and 12 out of 35 (34.2%) based on %Δt-MAD alone as true lead times. In the 14 cases where both NGS assay cutoffs were surpassed, 10 (71.4%) points were validated. These values indicated the higher sensitivity of %Δt-MAD compared to ΔVAF_mean_, while both showed similar specificity. Moreover, these results emphasized the potential of the combined NGS assays in increasing prediction confidence of early molecular progression. In total, we observed lead times in 19 out of 43 patients (44%), corresponding to 21 instances during TKI treatment, and one instance during chemotherapy. The median lead time to clinical progression was 88 days (range: 20–638 days). Fourteen of these 19 patients belonged to the group with detectable *ALK* alterations by tNGS (*n* = 22) as described above (Fig. 3b), indicating that calling lead times would be most relevant in patients or samples with high ctDNA content. Through this analysis, we also demonstrated the agreement and complementarity of tNGS (indicated by ΔVAF_mean_) and sWGS (indicated by %Δt-MAD) data in indicating molecular progression prior to standard radiographic assessment. Combined tNGS and sWGS data predicted lead times in 7 cases out of 22 (32%), tNGS data alone predicted lead times in five (23%) cases, and sWGS data alone was predictive in ten (45%) cases (Fig. 3c). No significant differences were found for the length of lead time predicted by either NGS assay, both independently and in combination (Fig. 3d). Lead time determination was also independent of therapy line (Fig. 3e). Time to molecular progression was significantly shorter in cases with lead time compared with clinical progression based on radiographic assessement (Fig. 3f). Lead times were longer for patients and/or therapy lines with longer duration of response (Fig. 3g). Out of 12 early molecular progression cases predicted by tNGS, 8 included *TP53* mutations (*TP53*^*mut*^) in the variants monitored longitudinally. Upon lead time comparison of *TP53*^*wt*^ versus *TP53*^*mut*^, we observed significantly shorter lead times in cases with *TP53* mutation (Fig. 3h).

### Relevance of *ALK* resistance mutations in early molecular progression events

We observed that early molecular progression identified through increased ΔVAF_mean_ were, in most cases, due to the cumulative contribution of known or probable *ALK* resistance mutations. Out of a total of 36 *ALK* mutations identified in our study, 20 overlapped with called lead times due to consistent and theshold-surpassing ΔVAF_mean_. From the remaining 16 mutations, 9 were detected at the time of radiologic progression (i.e., were not informative of lead time). The remaining seven instances represented mutations detected at the first sample analyzed for each patient (*n* = 3), which are likely clonal mutations present prior to therapy administration, and mutations arising under treatment (*n* = 4) whose associated ΔVAF_mean_ did not reach the threshold. In addition, the VAFs of these mutations declined before the next instance of radiologic progression. For emergent non-*ALK* mutations (*n* = 23), which included alterations in *BRAF*, *ERBB2*, *KRAS*, *MET*, *NRAS*, *RET*, and *TP53*, 13 mutation events were detected at the time of radiologic progression. From the remaining 10 mutations, two contributed to early molecular progression detection (i.e., their emergence contributed to ΔVAF_mean_ which exceeded the defined threshold), and 8 mutations emerged under treatment whose ΔVAF_mean_ did not reach the threshold, and subsequently the VAFs of these mutations declined before the next instance of radiologic progression. Altogether, our data indicate that resistance mutations, particularly known *ALK* resistance mutations, can indicate early molecular progression in many cases. However, the detection of lead time is more reliable in instances where such novel mutations arise in conjunction with increased ΔVAF_mean_, as applied in this work. Otherwise, detection of isolated and low allele frequency mutations pose the risk of identifying false-positive pseudo-progression events^34^.

### Longitudinal monitoring in representative ALK+ NSCLC patients

**Fig. 4:**
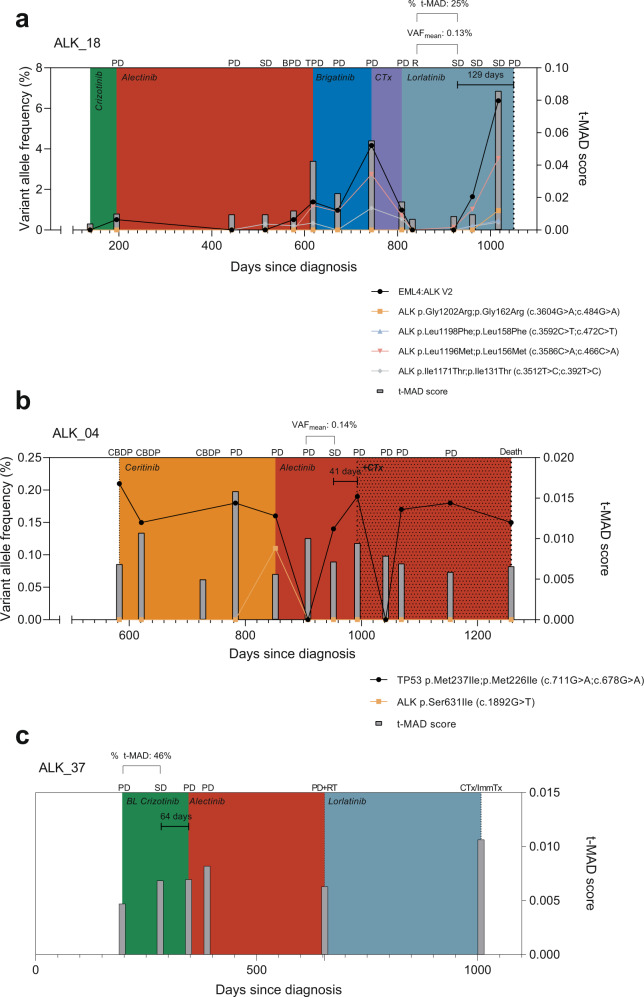
Representative cases illustrating early molecular progression as indicated by elevated ΔVAF_mean_ and %Δt-MAD. **a** Patient ALK_18 presented a case—during lorlatinib therapy—where both ΔVAF_mean_ and %Δt-MAD were informative of the lead time. Early molecular progression was apparent 129 days prior to clinical progression, as indicated by ΔVAF_mean_ of 0.13% and %Δt-MAD of 25%. Both values surpass the NGS metric thresholds identified in this study. **b** Patient ALK_04 depicts a case wherein ΔVAF_mean_ indicated early molecular progression during alectinib therapy. A ΔVAF_mean_ of 0.14% was calculated 41 days prior to clinical progression. **c** Patient ALK_37 emphasizes the relevance of untargeted NGS in ctDNA monitoring particularly in cases where genetic alterations remained undetected by tNGS. Here, %Δt-MAD of 46% was already apparent at a stable disease time point, 64 days prior to clinical progression. PD progressive disease, SD stable disease, BPD brain PD, TPD thoracic PD, R response, CBDP treatment continuation beyond disease progression, RT radiotherapy, CTx chemotherapy, ImmTx immunotherapy.

Representative cases of early molecular progression are illustrated in Fig. 4. Patient ALK_18 (Fig. 4a) depicts a case wherein both ΔVAF_mean_ and %Δt-MAD were in agreement in detecting early molecular progression at a stable disease point. During lorlatinib therapy, ALK_18 initially exhibited response as reflected by decreasing VAF and t-MAD score 65 days post initiation of treatment. Early molecular progression was detected 112 days post therapy initiation while the disease was evaluated as stable, and two subsequent sampling points showed continued increase of NGS metrics (i.e., ΔVAF_mean_ = 0.13%; %Δt-MAD = 25%) with stable disease until disease progression was observed. This point came 129 days after the onset of molecular progression. Patient ALK_04 (Fig. 4b) represents a case where ΔVAF_mean_ was instrumental in identifying a lead time during alectinib therapy. After disease progression on ceritinib (852 days since diagnosis), the therapy was switched to alectinib in which stable disease was observed 100 days post administration. However, molecular analysis at this point already revealed ΔVAF_mean_ of 0.14%, surpassing the threshold. Clinical progression was eventually observed 41 days later, supporting the result of ctDNA evaluation. Finally, patient ALK_37 (Fig. 4c) shows the utility of t-MAD monitoring particularly in the absence of detectable genetic alterations by tNGS. During crizotinib therapy, a %Δt-MAD increase of 46% was already apparent 86 days post treatment initiation, at which point the disease was still clinically stable. Disease progression occurred 64 days later, which led to a TKI therapy switch to alectinib. These case examples represent the 19 instances in our cohort where disease progression lead times were indicated by elevated levels of ctDNA through quantification of ΔVAF_mean_ and %Δt-MAD.

## Discussion

Due to the relatively long duration of treatment and emergence of actionable acquired resistance, therapy monitoring is especially important for TKI-treated NSCLC patients^35,36^. Early detection of disease progression could facilitate significant improvement in patient outcomes, as approximately 25–30% of NSCLC patients failing ALK or EGFR TKI miss subsequent therapy or chemotherapy due to rapid clinical deterioration^37,38^. Previously, shallow whole-genome sequencing has been applied to estimate global copy number changes from ctDNA through computation of the t-MAD score^28,39^. We further showed that this metric was associated with shorter overall survival in a subset of our ALK+ NSCLC cohort^27^. Here, we systematically examine whether combined tNGS and sWGS could also enable the detection of acquired resistance and disease progression earlier than radiological imaging.

Previously, an important challenge was the lack of a general consensus on how to measure ctDNA levels and response cutoffs. To date, multiple studies have used various ctDNA quantification methods including the maximum variant allele frequency^40^, the mean variant allele frequency^31,41^, and the total ctDNA concentration^32^. Similarly, response thresholds were typically established using a single time point after therapy baseline with arbitrary cutoff values^18,32,42^. Here, we employed ΔVAF_mean_ and the %Δt-MAD scores because these metrics can capture ctDNA changes regardless of how many SNV and/or CNV are present in the baseline sample. The use of ultra-deep targeted sequencing with optimized gene panels enables efficient surveillance of alteration hotspots of high clinical impact. These somatic changes are then used for early screening, treatment monitoring, and detection of residual disease. In parallel, untargeted sWGS approaches to liquid biopsy have been successful in other cancer entities^39,43,44^ and have been shown to reflect tumor status, even in cases without detectable SNVs^27^. Our data show that ΔVAF_mean_ offers better specificity than %Δt-MAD in discriminating progressive versus non-progressive disease. In contrast, %Δt-MAD showed increased sensitivity. These results suggest that ΔVAF_mean_ could be a more relevant ctDNA metric for clinical utility in the advanced setting where specificity is a critical performance assay criterion and greater ctDNA shedding occurs^44^, whereas %Δt-MAD could be applied at early disease stages. Nonetheless, residual false-positive and false-negative calls of disease status based on ctDNA evaluations could still take place due to tumors that shed minimal amounts of ctDNA; due to cases with apparent disagreement between molecular progression and imaging evaluations; and due to clearance of tumors leading to ctDNA shedding during earliest cycles of treatment.

In our study population, VAF_mean_ > 0 at therapy baseline predicted shorter therapy duration compared to cases without detected variants (VAF_mean_ = 0). This parallels findings demonstrating that ctDNA at baseline indicates poor prognosis in various cancers^18,27,45–47^. Pretreatment VAF levels have also been shown to be prognostic of treatment response with immune checkpoint inhibitors in patients with advanced solid tumors^31,48^. Pretreatment VAF_mean_ and maximum VAF were significantly inversely correlated with overall survival in NSCLC, urothelial cancer, microsatellite instable cancers, gastroesophageal cancer, and ovarian cancer^31^. Our results extend the potential of ctDNA detectability at therapy baseline as biomarkers for therapy response across various treatment lines in ALK+ NSCLC. Nonetheless, we recognize the marginal significance (*P* < 0.045) of our survival data. Variations in therapy sequence, therapy duration, and interpatient variabilities could be sources of confounding factors in the analysis. Each therapy line per patient was also treated as an independent case, discounting the potential effects of prior therapy.

A potential confounding factor in longitudinal monitoring lies in the assumption that visual evaluation of tumor radiographs accurately reflects tumor growth or progression^49^. In fact, there were sampling points attributed with clinically stable disease that showed consistently increasing levels of VAF_mean_ and/or t-MAD, which could indicate onset of disease progression below the limit of detection of radiographic modalities. By specifying numerical cutoffs for both NGS assays through ROC analysis, we were able to systematically identify such early molecular progression events in 44% of patients, across all therapy lines. This suggests that in this subset of our study cohort, ctDNA analysis exhibits higher sensitivity in detecting tumor progression than visual staging. It is also worthwhile to note that in majority of cases, early emergence of known or probable *ALK* mutations accounted for increased ΔVAF_mean_ that resulted in lead time identification. This emphasizes the sensitivity of ctDNA analysis in identifying potentially actionable mutations early in the disease course. From the tumor biology standpoint, such cases reflect the dynamic clonal evolution within the tumor that could potentially be missed by tissue re-biopsies. Nonetheless, there were also mutations—both *ALK* and non-*ALK*—that were inconsistent or sporadic throughout the longitudinal course. Using ΔVAF_mean_ as a cumulative metric of ctDNA levels minimizes identification of such false-positive pseudo-molecular progression events that could have arisen from stochastic sampling issues^50^.

While both tNGS and sWGS were able to independently call early molecular progression events, we also present evidence that a combined evaluation increases the confidence in predicting lead times by approximately 50%, corroborating our previous observation^27^ that untargeted sWGS complements tNGS. Moreover, there was a positive correlation between the length of predicted lead time and durability of response to therapy. Therefore, molecular monitoring for earlier detection of disease progression could be even more useful for patients treated with newer ALK inhibitors, namely alectinib, brigatinib, or lorlatinib, whose progression-free survival extends well beyond two years, in contrast to 10–12 months under crizotinib^8–10^. This approach also limits the risk due to higher cumulative radiation exposure from regular CTs^51^. Interestingly, we further observed that lead times involving mutations in *TP53* were shorter compared to *TP53* wild types. This implies that the emergence of *TP53* alterations during therapy course portends a swifter progression of disease, as previously noted in ALK+ NSCLC^52–54^. Ideally, early detection of molecular progression could prompt clinicians to halt ineffective treatments, avoiding potential side effects and added financial burden, and also facilitate an earlier therapy switch, while the patients are still capable of receiving next-line therapies^38^. Such a strategy could be particularly useful for cases with higher risk, such as those with *TP53* mutations and/or the *EML4-ALK* variant 3 (refs. ^53,55^). This approach is complementary to monitoring of early ctDNA changes after therapy initiation, which have been shown to predict therapy durability^44^.

The main limitations of our study are related to its retrospective design, the relatively small patient number, and the lack of a separate validation cohort. Our findings should ideally be confirmed in a larger, prospective study with strictly defined sampling and imaging intervals. The variants by tNGS were also limited to the genes included in the panels utilized, thus other genetic alterations in ctDNA were not evaluated.

Our work demonstrates the applicability of a combined targeted and untargeted NGS analysis of cfDNA for longitudinal monitoring and identification of early molecular progression in ALK+ NSCLC. We established ΔVAF_mean_ and %Δt-MAD as quantifiable metrics for ctDNA levels and determined thresholds to enable prediction of disease progression and identification of early molecular progression. Currently, the development of robust criteria assessing ctDNA dynamics that correlate with clinical status such as disease progression, therapy response, and therapy resistance remains challenging partly due to stochastic sampling issues^50^, and the spectrum of ctDNA shedding tendencies of different malignancies. Aside from ctDNA, it should also be noted that other cell-free markers have the capacity to detect progression and predict disease recurrence. In NSCLC, for example, miRNA signatures in the plasma have been shown to associate with higher risk for progression^56,57^, while a separate panel could predict survival in squamous cell carcinoma patients^56^. Serum carcinoembryonic antigen (CEA) is also associated with distinct cancer progression profiles and carries predictive information of risk recurrence in NSCLC^58,59^. Oncogenic mRNA markers in plasma have also shown clinical utility in NSCLC^60,61^. Thus, in addition to genomic alterations in ctDNA, future investigations of other plasma solutes including proteins, miRNAs, mRNAs, and the epigenomic properties of ctDNA (e.g., methylation, nucleosome positioning) will advance the potential of liquid biopsy in complementing imaging technologies in improving personalized patient management and efficient therapy decisions.

## Methods

### Patients and sample collection

The study was approved by the ethics committees of Heidelberg University (S-270/2001, S-296/2016) and Lübeck University (AZ 12-238). Written informed consent was obtained from all study participants. Newly diagnosed cases were screened for the presence of an *ALK* alteration in tissues by fluorescence in situ hybridization (FISH, ZytoLight SPEC ALK probe, ZytoVision GmbH, Bremerhaven, Germany) and reverse-transcription polymerase chain reaction until 2015, or by immunohistochemistry (D5F3 clone, Roche, Mannheim, Germany) and RNA-based next-generation sequencing (NGS, Thermo Fisher Lung Cancer Fusion Panel, Waltham, MA, USA) thereafter, as previously described^12^. Peripheral blood was prospectively drawn through venipuncture at each outpatient visit from 84 ALK+ patients with metastatic NSCLC under TKI therapy at Thoraxklinik Heidelberg and Lungenclinic Grosshansdorf, Germany^62^. In total, 412 longitudinal blood samples were collected and plasma was isolated within 1 h of blood draw, using the double spin method as previously described^27^. Samples were stored at −80 °C in the Lung Biobank Heidelberg/BMBH until further processing.

Since our study was focused on longitudinal therapy monitoring, we limited the scope of our analysis only on patients with at least four evaluable plasma samples throughout their treatment course. Using these criteria, we excluded 41 patients (69 plasma samples). Ultimately, 43 metastatic ALK+ patients with corresponding 343 plasma samples were considered for NGS analysis (Fig. 1a). The characteristics of these patients are summarized in Table 1. Clinical data and the results of routine radiographic assessments (every 8–12 weeks) using chest/abdominal CT and brain MRI were collected through a review of patient records with a cutoff on September 15, 2020.

### cfDNA isolation

The AVENIO cfDNA Isolation Kit (Roche Diagnostics) was used to isolate cfDNA from 2 mL of patient plasma, following the manufacturer’s protocol. The characteristic mononucleosomal molecular weight profile (160–200 bp) of the purified cfDNA was assessed using the Bioanalyzer 2100 High Sensitivity DNA Kit (Agilent Technologies). The cfDNA was quantified using the Qubit dsDNA High Sensitivity Kit (Thermo Fisher).

### cfDNA NGS assays

Sequencing libraries for capture-based targeted sequencing were generated using the AVENIO ctDNA Library Preparation Kit with either the Targeted or Surveillance Panel (Roche Diagnostics), as previously described^27^. Equimolar 16-plex enriched library pools were sequenced on the Illumina NextSeq 550 platform with the High-Output Kit V2 (2 × 150 bp). The resulting raw BCL files were processed using the AVENIO ctDNA analysis software (Roche Diagnostics, version 2.0.0). The proprietary analysis pipeline applied by the software is adapted from the CAPP-Seq workflow with integrated digital suppression^63,64^. Called variants with variant allele frequencies (VAFs) ≥30% were classified as germline mutations and were excluded from subsequent analyses. Only genes common to both Targeted and Surveillance panels (Supplementary Table 3) were compared for all longitudinal assessments, with a VAF calling threshold of 0.01%, facilitated by an average of 4100× unique target coverage as previously reported^27^. For sWGS, sequencing libraries were prepared using the KAPA HyperPrep Kit with KAPA Dual-Indexed Adapters for Illumina platforms, as previously described^27^ using 1–2.5 ng cfDNA as input. Pooled multiplexes of 48–67 equimolar libraries were sequenced on the Illumina HiSeq 4000 platform (2 × 100 bp). To determine genome-wide copy number variations (CNVs) and calculate the t-MAD scores, raw sWGS sequencing data were processed and analyzed as previously described^27^. Briefly, automated sequence quality control and alignment were performed using the One Touch Pipeline^65^. Genome-wide copy number profiles and tumor fractions were estimated using ichorCNA implemented in R (version 3.3.1). T-MAD scores were computed using a 1-Mb bin size and without fragment size selection^27^.

### Genomic alteration and t-MAD score determination

Somatic SNVs and indels detected by the AVENIO ctDNA analysis software were used to calculate the mean variant allele frequency (VAF_mean_) per sampling point. This value is the sum of all VAFs divided by the number of all mutations at each time point to account for polyclonality^17^. The change in mean VAF (ΔVAF_mean_) was the difference in VAF_mean_ between two successive time points as shown by Eqs. (1) and (2).

1 $$\Delta {\mathrm {VAF}}_{{\mathrm {mean}}} = \overline {{\mathrm {VAF}}_2} - \overline {{\mathrm {VAF}}_1}$$

where

2 $$\overline {{\mathrm {VAF}}} = \frac{{\mathop {\sum }\nolimits_{i = 1}^n {\mathrm {VAF}}_i}}{n}$$

Here, *n* represents the number of detected variants. If *n* = 0, or no variant was detected, VAF_mean_ was set to 0. Positive ΔVAF_mean_ values indicate increasing VAF_mean_. In parallel, the change in t-MAD (%Δt-MAD) was calculated, in percentage, as the difference between two successive t-MAD scores, divided by the t-MAD measured at the previous time point as shown by Eq. (3).

3 $${{{{{\mathrm{\% }}}}}}\Delta t - {\mathrm {MAD}} = \left( {\frac{{{\mathrm t} {\mbox{-}} {\mathrm {MAD}}_2 - {\mathrm t} \mbox{-} {\mathrm {MAD}}_1}}{{{\mathrm t} \mbox{-} {\mathrm {MAD}}_1}}} \right) \times 100{{{{{\mathrm{\% }}}}}}$$

ΔVAF_mean_ and %Δt-MAD thresholds for disease progression were derived using the respective receiver operating characteristic (ROC) curve across measurement time points with progressive disease accompanied by therapy change (PD_therapy change_) as the event of interest. The annotation of PD_therapy change_ was verified manually based on radiological imaging according to RECIST criteria v1.1 (ref. ^66^), as well as information about patient treatment from the medical records. Area under the curve (AUC) values were generated using GraphPad Prism 9 (GraphPad software, La Jolla California USA), while cutoff values were identified using the Youden Index, which maximizes the sensitivity and specificity of the NGS metric (i.e., ΔVAF_mean_ and %Δt-MAD)^67^. Seven patients (ALK_22, 37, 57, 59, 60, 66, 105) were excluded from ΔVAF_mean_ measurements due to the absence of detectable variants in all plasma DNA samples. In total, 162 measurements of ΔVAF_mean_ and 184 measurements of %Δt-MAD were used for ROC curve analyses (Supplementary Tables 4 and 5).

We defined early molecular progression as a change in either NGS metric (i.e., ΔVAF_mean_ or %Δt-MAD) surpassing the threshold value defined from the ROC curve analysis, from a point where the tumor was radiographically stable according to RECIST to a later progression point. The lead time was defined as the number of days between the first observation of molecular progression until the point of an initial clinical progression as per radiographic assessment based on RECIST.

### Statistical analysis and data visualization

Survival data were analyzed using the log-rank test. Lead time comparisons were performed using the Mann–Whitney *U* test or one-way ANOVA, as labeled in the graphs. Pairwise comparison of days to progression was performed using the Wilcoxon’s paired test. Since the primary aim of this study was to establish the relationship between ctDNA changes and disease progression, samples from different progression time points of the same patient were analyzed as independent, in order to account for the fact that several factors can influence liquid biopsy positivity differently in each sample of the same patient (e.g. progression site, progression rate, tumor volume, preceding treatment), and that these factors act similarly across different patients. Statistical analyses, including ROC curves, and relevant graphs were performed and generated, respectively, using GraphPad Prism 9.

### Reporting summary

Further information on research design is available in the Nature Research Reporting Summary linked to this article.

## Supplementary information

Supplementary Information

Reporting Summary

## Data Availability

All sequencing data supporting the findings in this study are deposited in the European Genome-phenome Archive (EGA) under accession number EGAS00001005327.
