# Supplementary material for: Early identification of disease progression in ALK-rearranged lung cancer using circulating tumor DNA analysis
Source: NPJ Precis Oncol. 2021 Dec 7;5:100. doi: 10.1038/s41698-021-00239-3 (PMC8651695; doi:10.1038/s41698-021-00239-3)
Supplement: Supplementary file 1 — Supplementary Information [file 41698_2021_239_MOESM1_ESM.pdf]

Early identification of disease progression in *ALK*-rearranged lung cancer using circulating tumor DNA analysis

Supplementary Information

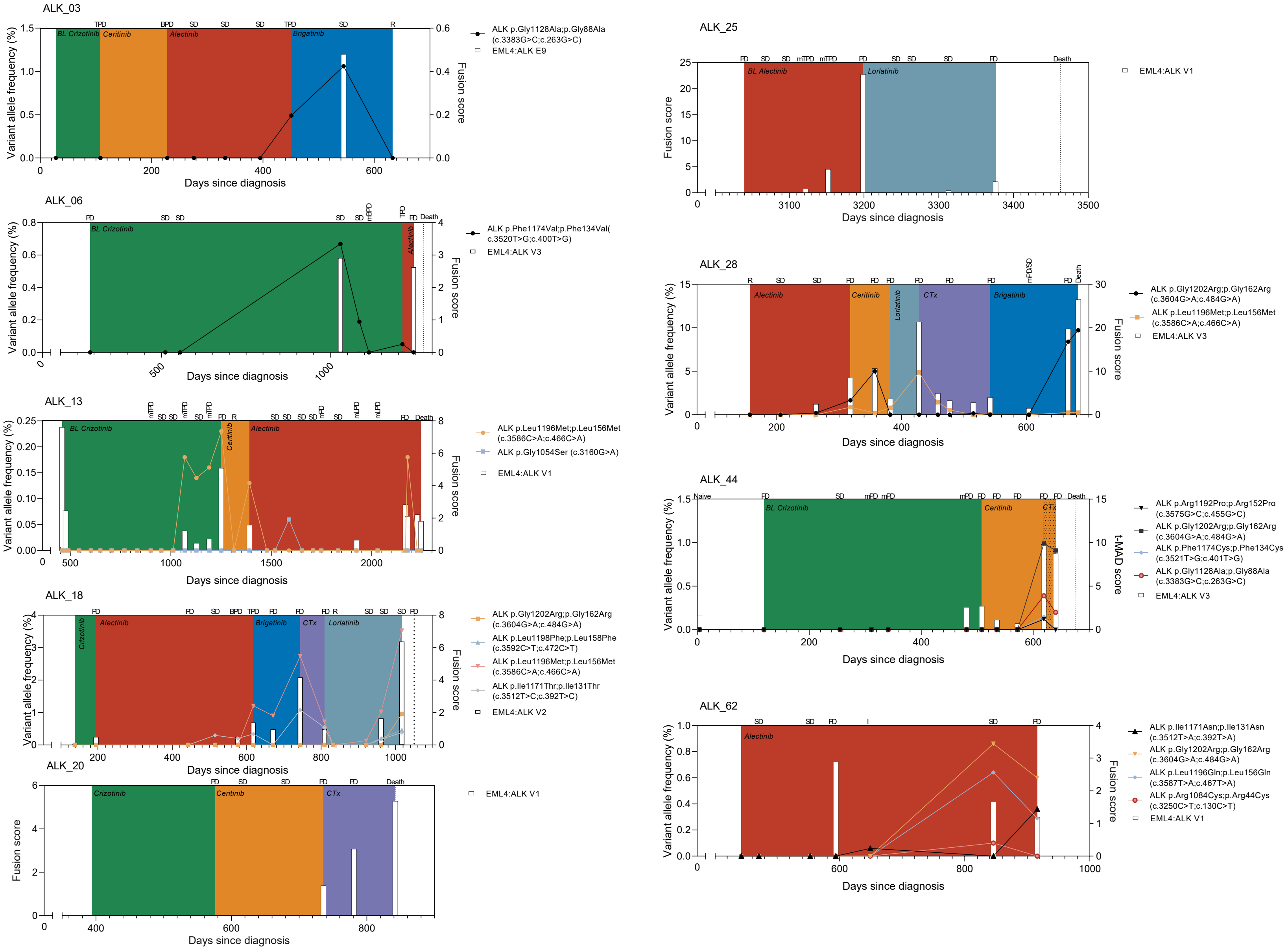

Supplementary figure 1. ctDNA kinetics of representative patients showing the concordance of *ALK* alterations with clinical course across therapy lines. Only *ALK* alterations are shown here for clarity. PD: progressive disease. SD: stable disease. BPD: brain PD. mBPD: mild BPD. TPD: thoracic PD. mTPD: mild TPD. R: response. I: improvement. CTx: chemotherapy.

Supplementary Table 1. *ALK* mutations and rearrangements determined from targeted NGS of ctDNA.

**Patient**	**Fusion detected**	**Mutations detected**			
		**Nomenclature**		**Clinical relevance**	**Reference (DOI)**
**ALK_03**	EML4;ALK	p.Gly1128Ala;p.Gly88Ala	ALK c.3383G>C;c.263G>C	known resistance mutation	10.1016/j.lungcan.2018.07.004
**ALK_04**	ND	p.Ser631Ile	ALK c.1892G>T	predicted pathogenic (probable resistance mutation)	10.1093/nar/gky1015
**ALK_05**	EML4;ALK	ND	ND		
**ALK_06**	EML4;ALK	p.Phe1174Val;p.Phe134Val	ALK c.3520T>G;c.400T>G	known resistance mutation	10.1097/JTO.0000000000000094
**ALK_08**	EML4;ALK	ND	ND		
**ALK_12**	ND	p.Gly1269Ala;p.Gly229Ala	ALK c.3806G>C;c.686G>C	known resistance mutation	10.1158/2159-8290.CD-16-0596
**ALK_12**		p.Phe1174Leu;p.Phe134Leu	ALK c.3522C>G;c.402C>G	known resistance mutation	10.1158/2159-8290.CD-16-0596
**ALK_12**		p.Phe1174Leu;p.Phe134Leu	ALK c.3522C>A;c.402C>A	known resistance mutation	10.1158/2159-8290.CD-16-0596
**ALK_12**		p.Phe1174Cys;p.Phe134Cys	ALK c.3521T>G;c.401T>G	known resistance mutation	10.1158/2159-8290.CD-16-0596
**ALK_12**		p.Phe1174Val;p.Phe134Val	ALK c.3520T>G;c.400T>G	known resistance mutation	10.1158/2159-8290.CD-16-0596
**ALK_12**		p.Phe1174Leu;p.Phe134Leu	ALK c.3520T>C;c.400T>C	known resistance mutation	10.1158/2159-8290.CD-16-0596
**ALK_12**		p.Ile1171Thr;p.Ile131Thr	ALK c.3512T>C;c.392T>C	known resistance mutation	10.1158/2159-8290.CD-16-0596
**ALK_12**		p.Leu1204Leu;p.Leu164Leu	ALK c.3612C>G;c.492C>G	silent mutation	
**ALK_12**		p.Arg1192Pro;p.Arg152Pro	ALK c.3575G>C;c.455G>C	known resistance mutation	10.18632/oncotarget.8173
**ALK_12**		p.Gly1269Ala;p.Gly229Ala	ALK c.3806G>C;c.686G>C	known resistance mutation	10.1111/1759-7714.13299
**ALK_13**	EML4;ALK	p.Leu1196Met;p.Leu156Met	ALK c.3586C>A;c.466C>A	known resistance mutation	10.1158/2159-8290.CD-16-0596
**ALK_13**		p.Gly1054Ser	ALK c.3160G>A	predicted pathogenic (probable resistance mutation)	10.1093/nar/gky1015
**ALK_16**	EML4;ALK	ND	ND		
**ALK_18**	EML4;ALK	p.Gly1202Arg;p.Gly162Arg	ALK c.3604G>A;c.484G>A	known resistance mutation	10.1158/2159-8290.CD-16-0596
**ALK_18**		p.Leu1198Phe;p.Leu158Phe	ALK c.3592C>T;c.472C>T	known resistance mutation	10.1056/NEJMoa1508887
**ALK_18**		p.Leu1196Met;p.Leu156Met	ALK c.3586C>A;c.466C>A	known resistance mutation	10.1158/2159-8290.CD-16-0596
**ALK_18**		p.Ile1171Thr;p.Ile131Thr	ALK c.3512T>C;c.392T>C	known resistance mutation	10.1158/2159-8290.CD-16-0596
**ALK_20**	EML4;ALK	ND	ND		
**ALK_21**	ND	p.Leu1044Leu	ALK c.3132C>T	silent mutation	
**ALK_21**		p.Gly1269Ala;p.Gly229Ala	ALK c.3806G>C;c.686G>C	known resistance mutation	10.1158/2159-8290.CD-16-0596
**ALK_25**	EML4;ALK	ND	ND		
**ALK_28**	EML4;ALK	p.Gly1202Arg;p.Gly162Arg	ALK c.3604G>A;c.484G>A	known resistance mutation	10.1158/2159-8290.CD-16-0596
**ALK_28**		p.Leu1196Met;p.Leu156Met	ALK c.3586C>A;c.466C>A	known resistance mutation	10.1158/2159-8290.CD-16-0596

**ALK_30**	ND	p.Gly1202Arg;p.Gly162Arg	ALK c.3604G>A;c.484G>A	known resistance mutation	10.1158/2159-8290.CD-16-0596
**ALK_32**	EML4;ALK	ND	ND		
**ALK_40**	EML4;ALK<br>RAD51AP2;ALK	ND	ND		
**ALK_44**	EML4;ALK	p.Gly1202Arg;p.Gly162Arg	ALK c.3604G>A;c.484G>A	known resistance mutation	10.1158/2159-8290.CD-16-0596
**ALK_44**		p.Arg1192Pro;p.Arg152Pro	ALK c.3575G>C;c.455G>C	known resistance mutation	10.18632/oncotarget.8173
**ALK_44**		p.Phe1174Cys;p.Phe134Cys	ALK c.3521T>G;c.401T>G	known resistance mutation	10.1158/2159-8290.CD-16-0596
**ALK_44**		p.Gly1128Ala;p.Gly88Ala	ALK c.3383G>C;c.263G>C	known resistance mutation	10.1016/j.lungcan.2018.07.004
**ALK_101**	ND	p.Gly1269Ala;p.Gly229Ala	ALK c.3806G>C;c.686G>C	known resistance mutation	10.1158/2159-8290.CD-16-0596
**ALK_101**		p.Gly1202Arg;p.Gly162Arg	ALK c.3604G>A;c.484G>A	known resistance mutation	10.1158/2159-8290.CD-16-0596
**ALK_48**	EML4;ALK	ND	ND		
**ALK_56**	EML4;ALK	p.Leu1196Met;p.Leu156Met	ALK c.3586C>A;c.466C>A	known resistance mutation	10.1158/2159-8290.CD-16-0596
**ALK_62**	EML4;ALK	p.Ile1171Asn;p.Ile131Asn	ALK c.3512T>A;c.392T>A	known resistance mutation	10.1158/2159-8290.CD-16-0596
**ALK_62**		p.Gly1202Arg;p.Gly162Arg	ALK c.3604G>A;c.484G>A	known resistance mutation	10.1158/2159-8290.CD-16-0596
**ALK_62**		p.Leu1196Gln;p.Leu156Gln	ALK c.3587T>A;c.467T>A	known resistance mutation	10.1158/1541-7786.MCR-12-0569
**ALK_62**		p.Arg1084Cys;p.Arg44Cys	ALK c.3250C>T;c.130C>T	predicted pathogenic (probable resistance mutation)	doi.org/10.1093/nar/gky1015
**ALK_104**	ND	p.Gly893Arg	ALK c.2677G>A	predicted pathogenic (probable resistance mutation)	doi.org/10.1093/nar/gky1015

ND: not detectable

Supplementary table 2. Genetic alterations emergent* at disease progression.

Alteration	Patient	VAF at PD†	Therapy line	Protein change	Short-hand
**ALK c.1892G>T**	ALK_04	0.11%	Ceritinib	p.Ser631Ile	S631I
**ALK c.3250C>T;c.130C>T**	ALK_62	0.10%	Alectinib	p.Arg1084Cys;p.Arg44Cys	R1084C
**ALK c.3383G>C;c.263G>C**	ALK_03	0.49%	Alectinib	p.Gly1128Ala;p.Gly88Ala	G1128A
**ALK c.3383G>C;c.263G>C**	ALK_44	0.39%	Ceritinib	p.Gly1128Ala;p.Gly88Ala	G1128A
**ALK c.3512T>A;c.392T>A**	ALK_62	0.36%	Alectinib	p.Ile1171Asn;p.Ile131Asn	I1171N
**ALK c.3512T>C;c.392T>C**	ALK_12	0.03%	Crizotinib	p.Ile1171Thr;p.Ile131Thr	I1171T
**ALK c.3520T>C;c.400T>C**	ALK_12	0.05%	Crizotinib	p.Phe1174Leu;p.Phe134Leu	F1174L
**ALK c.3520T>G;c.400T>G**	ALK_12	0.02%	Crizotinib	p.Phe1174Val;p.Phe134Val	F1174V
**ALK c.3521T>G;c.401T>G**	ALK_12	0.08%	Crizotinib	p.Phe1174Cys;p.Phe134Cys	F1174C
**ALK c.3521T>G;c.401T>G**	ALK_44	0.15%	Ceritinib	p.Phe1174Cys;p.Phe134Cys	F1174C
**ALK c.3522C>A;c.402C>A**	ALK_12	0.24%	Crizotinib	p.Phe1174Leu;p.Phe134Leu	F1174L
**ALK c.3522C>G;c.402C>G**	ALK_12	0.88%	Crizotinib	p.Phe1174Leu;p.Phe134Leu	F1174L
**ALK c.3575G>C;c.455G>C**	ALK_12	0.03%	Crizotinib	p.Arg1192Pro;p.Arg152Pro	R1192P
**ALK c.3575G>C;c.455G>C**	ALK_44	0.12%	Ceritinib	p.Arg1192Pro;p.Arg152Pro	R1192P
**ALK c.3586C>A;c.466C>A**	ALK_13	0.18%	Crizotinib	p.Leu1196Met;p.Leu156Met	L1196M
**ALK c.3586C>A;c.466C>A**	ALK_13	0.18%	Alectinib	p.Leu1196Met;p.Leu156Met	L1196M
**ALK c.3586C>A;c.466C>A**	ALK_18	1.21%	Alectinib	p.Leu1196Met;p.Leu156Met	L1196M
**ALK c.3586C>A;c.466C>A**	ALK_28	0.87%	Alectinib	p.Leu1196Met;p.Leu156Met	L1196M
**ALK c.3586C>A;c.466C>A**	ALK_13	0.13%	Ceritinib	p.Leu1196Met;p.Leu156Met	L1196M
**ALK c.3604G>A;c.484G>A**	ALK_28	8.41%	Alectinib	p.Gly1202Arg;p.Gly162Arg	G1202R
**ALK c.3604G>A;c.484G>A**	ALK_44	0.99%	Ceritinib	p.Gly1202Arg;p.Gly162Arg	G1202R
**ALK c.3604G>A;c.484G>A**	ALK_28	8.41%	Brigatinib	p.Gly1202Arg;p.Gly162Arg	G1202R
**ALK c.3612C>G;c.492C>G**	ALK_12	0.06%	Crizotinib	p.Leu1204Leu;p.Leu164Leu	L1204L
**ALK c.3806G>C;c.686G>C**	ALK_12	0.43%	Crizotinib	p.Gly1269Ala;p.Gly229Ala	G1269A
**ALK;EML4 fusion**	ALK_18	0.50752688	Crizotinib		
**ALK;EML4 fusion**	ALK_44	2.44638927	Crizotinib		

**ALK;EML4 fusion**	ALK_13	2.23385	Alectinib		
**ALK;EML4 fusion**	ALK_18	0.50770308	Alectinib		
**ALK;EML4 fusion**	ALK_25	0.7276359	Alectinib		
**ALK;EML4 fusion**	ALK_62	2.88461539	Alectinib		
**ALK;EML4 fusion**	ALK_13	1.57927	Ceritinib		
**ALK;EML4 fusion**	ALK_20	1.38	Ceritinib		
**ALK;EML4 fusion**	ALK_06	2.632088	Alectinib		
**BRAF c.1405G>C**	ALK_44	0.11%	Ceritinib	p.Gly469Arg	G469R
**EGFR CNV**	ALK_05	8.83	Crizotinib		
**EGFR CNV**	ALK_13	4.544	Crizotinib		
**EGFR CNV**	ALK_44	3.39224386	Crizotinib		
**EGFR CNV**	ALK_13	3.03324366	Alectinib		
**EGFR CNV**	ALK_25	3.85624385	Alectinib		
**EGFR CNV**	ALK_62	6.879243851; 0.541243732	Alectinib		
**EGFR CNV**	ALK_28	8.15124	Brigatinib		
**EGFR CNV**	ALK_30	4.30911446	Brigatinib		
**EGFR CNV**	ALK_28	0.96424	Lorlatinib		
**ERBB2 c.1218C>T;c.1173C>T**	ALK_13	0.20%	Alectinib	p.Ile406Ile;p.Ile406Ile;p.Ile391Ile	I406=
**ERBB2 c.1976T>A;c.1976T>A;c.1931T>A**	ALK_27	0.61%	Alectinib	p.Val659Asp;p.Val659Asp;p.Val644Asp	V659D
**ERBB2 c.2313_2324dupATACGTGATGGC; c.2268_2279dupATACGTGATGGC**	ALK_13	1.20%	Crizotinib	p.Ala775_Gly776insTyrValMetAla; p.Ala775_Gly776insTyrValMetAla; p.Ala760_Gly761insTyrValMetAla	
**KRAS c.436G>A**	ALK_12	0.16%	Crizotinib	p.Ala146Thr;p.Ala146Thr	A146T
**KRAS c.436G>C**	ALK_10	0.07%	Crizotinib	p.Ala146Pro;p.Ala146Pro	A146P
**MET c.1690G>A**	ALK_21	0.28%	Alectinib	p.Ala564Thr;p.Ala564Thr;p.Ala564Thr	A564T
**MET c.1690G>A**	ALK_05	0.09%	Crizotinib	p.Ala564Thr;p.Ala564Thr;p.Ala564Thr	A564T

**MET c.3743A>G;c.3689A>G**	ALK_05	0.02%	Crizotinib	p.Tyr1248Cys;p.Tyr1230Cys	Y1248C
**MET CNV**	ALK_05	7.78	Crizotinib		
**MET CNV**	ALK_13	4.796	Crizotinib		
**MET CNV**	ALK_18	0.29224363	Crizotinib		
**MET CNV**	ALK_13	1.97511435	Alectinib		
**MET CNV**	ALK_25	2.7981143	Alectinib		
**MET CNV**	ALK_28	14.1251	Brigatinib		
**MET CNV**	ALK_30	11.5672436	Brigatinib		
**NRAS c.34G>T**	ALK_44	0.03%	Ceritinib	p.Gly12Cys	G12C
**NRAS c.38G>T**	ALK_20	0.03%	CTx	p.Gly13Val	G13V
**RET c.1907C>T;c.1907C>T**	ALK_35	0.12%	Crizotinib	p.Thr636Met;p.Thr636Met	T636M
**TP53 c.374C>G**	ALK_20	1%	Ceritinib	p.Thr125Arg;p.Thr125Arg;p.Thr125Arg;p.Thr125Arg	T125R
**TP53 c.581T>C;c.548T>C**	ALK_05	0.18%	Crizotinib	p.Leu194Pro;p.Leu194Pro;p.Leu194Pro;p.Leu194Pro;p.Leu183Pro	L194P
**TP53 c.586C>T;c.553C>T**	ALK_13	2.02%	Alectinib	p.Arg196*;p.Arg196*;p.Arg196*;p.Arg196*;p.Arg185*	R196*
**TP53 c.641A>G;c.608A>G**	ALK_13	0.07%	Ceritinib	p.His214Arg;p.His214Arg;p.His214Arg;p.His214Arg;p.His203Arg	H214R
**TP53 c.659A>G;c.626A>G**	ALK_56	1.92%	Alectinib	p.Tyr220Cys;p.Tyr220Cys;p.Tyr220Cys;p.Tyr220Cys;p.Tyr209Cys	Y220C
**TP53 c.715A>G;c.682A>G**	ALK_20	0.12%	Crizotinib	p.Asn239Asp;p.Asn239Asp;p.Asn239Asp;p.Asn239Asp;p.Asn228Asp	N239D
**TP53 c.722C>G;c.689C>G**	ALK_03	0.17%	Alectinib	p.Ser241Cys;p.Ser241Cys;p.Ser241Cys;p.Ser241Cys;p.Ser230Cys	S241C
**TP53 c.730G>T;c.697G>T**	ALK_03	0.10%	Crizotinib	p.Gly244Cys;p.Gly244Cys;p.Gly244Cys;p.Gly244Cys;p.Gly233Cys	G244C
**TP53 c.818G>T;c.785G>T**	ALK_56	0.19%	Alectinib	p.Arg273Leu;p.Arg273Leu;p.Arg273Leu;p.Arg262Leu	R273L
**TP53 c.920-2A>G;c.887-2A>G**	ALK_28	5.19%	Alectinib	N/A	
**TP53 c.994-2A>G;c.*101-2A>G; c.*13-2A>G;c.961-2A>G**	ALK_20	0.30%	CTx	N/A	

*Genetic alterations detected in samples collected at clinical progression points which were not detected in previous sampling points within the course of a therapy line.

†Fusion score was quantified as the number of paired reads spanning the fusion breakpoint divided by the median sequencing depth. The CNV score reported here is the output from the Avenio analysis pipeline, which is defined as a statistical summary of a called amplification accounting the log2ratio to normal copy number.

Supplementary table 3. Genes included in the targeted sequencing panels.

Gene	Exon	SNV	Indel	Fusion	CNV
ALK	4, 12, 15, 20, 21, 22, 23, 24, 25, 28	x	x	x	
APC	6, 7, 9, 12, 14, 16	x	x		
BRAF	11, 15	x	x		
BRCA1	all	x			
BRCA2	all	x			
DPYD	all	x			
EGFR	all	x	x		x
ERBB2	all	x	x		x
KIT	8, 9, 11, 13, 14, 17	x	x		
KRAS	all	x			
MET	all	x	x		x
NRAS	2, 3	x			
PDGFRA	11, 12, 14, 15, 16, 18	x			
RET	11, 16	x		x	
ROS1	38	x		x	
TP53	all	x	x		

Supplementary table 4. %Δt-MAD used for ROC analyses.

ALK_ID	%Δt-MAD	Clinical status
ALK_03	25%	PDTC
ALK_03	157%	PDTC
ALK_03	-62%	SD
ALK_03	-1%	SD
ALK_03	-15%	SD
ALK_03	197%	PDTC
ALK_03	-26%	SD
ALK_03	-34%	SD
ALK_04	-29%	SD
ALK_04	32%	PDTC
ALK_05	-8%	SD
ALK_05	-43%	SD
ALK_05	129%	SD
ALK_05	-45%	SD
ALK_06	77%	SD
ALK_06	20%	SD
ALK_06	58%	SD
ALK_06	-79%	SD
ALK_06	12%	PDTC
ALK_07	-15%	SD
ALK_07	810%	PDTC
ALK_07	-84%	SD
ALK_07	-13%	SD
ALK_08	42%	SD
ALK_08	-28%	SD
ALK_08	-18%	SD
ALK_10	-31%	SD
ALK_10	-19%	SD
ALK_12	12%	SD
ALK_12	-21%	SD
ALK_12	8%	SD
ALK_12	197%	PDTC
ALK_12	-76%	SD
ALK_12	24%	SD
ALK_12	78%	SD
ALK_12	24%	SD
ALK_12	-14%	SD
ALK_12	26%	SD
ALK_13	-67%	SD
ALK_13	157%	SD
ALK_13	9%	SD
ALK_13	-27%	SD
ALK_13	-3%	SD
ALK_13	21%	SD

ALK_13	93%	SD
ALK_13	-8%	SD
ALK_13	-21%	SD
ALK_13	51%	PDTC
ALK_13	-64%	SD
ALK_13	115%	SD
ALK_13	16%	SD
ALK_13	2%	SD
ALK_13	39%	SD
ALK_13	-50%	SD
ALK_13	2%	SD
ALK_15	13%	SD
ALK_15	64%	SD
ALK_15	8%	PDTC
ALK_15	-26%	SD
ALK_15	135%	SD
ALK_15	-18%	SD
ALK_15	-17%	SD
ALK_16	45%	PDTC
ALK_16	-22%	SD
ALK_18	155%	PDTC
ALK_18	0%	SD
ALK_18	257%	PDTC
ALK_18	145%	PDTC
ALK_18	-61%	SD
ALK_18	25%	SD
ALK_18	13%	SD
ALK_18	791%	SD
ALK_20	3%	PDTC
ALK_20	-25%	SD
ALK_20	49%	SD
ALK_21	9%	SD
ALK_21	57%	PDTC
ALK_21	24%	PDTC
ALK_22	44%	SD
ALK_22	0%	SD
ALK_23	45%	SD
ALK_23	1%	SD
ALK_23	19%	SD
ALK_23	-21%	SD
ALK_23	50%	SD
ALK_23	-23%	SD
ALK_24	-15%	SD
ALK_24	15%	SD
ALK_24	64%	SD
ALK_24	-16%	SD
ALK_24	28%	SD

ALK_24	-5%	SD
ALK_24	-3%	SD
ALK_24	-8%	SD
ALK_25	39%	SD
ALK_25	-19%	SD
ALK_25	99%	PDTC
ALK_25	-51%	SD
ALK_25	34%	SD
ALK_25	-29%	SD
ALK_26	40%	SD
ALK_26	98%	SD
ALK_26	-54%	SD
ALK_26	77%	SD
ALK_26	-29%	SD
ALK_27	37%	PDTC
ALK_27	-44%	SD
ALK_27	-29%	SD
ALK_27	-25%	SD
ALK_27	13%	SD
ALK_28	-22%	SD
ALK_28	13%	SD
ALK_28	436%	PDTC
ALK_28	609%	PDTC
ALK_28	11%	PDTC
ALK_28	17%	PDTC
ALK_29	-9%	SD
ALK_29	34%	SD
ALK_30	23%	SD
ALK_30	300%	SD
ALK_30	-89%	SD
ALK_30	514%	PDTC
ALK_30	-29%	SD
ALK_30	-18%	SD
ALK_30	33%	SD
ALK_30	19%	SD
ALK_30	12%	PDTC
ALK_30	764%	PDTC
ALK_30	2%	PDTC
ALK_31	28%	SD
ALK_32	-51%	SD
ALK_32	8%	PDTC
ALK_32	21%	SD
ALK_32	-61%	SD
ALK_34	19%	SD
ALK_34	-12%	SD
ALK_34	131%	SD
ALK_34	-3%	SD

ALK_35	-8%	SD
ALK_35	67%	PDTC
ALK_35	-30%	SD
ALK_37	46%	SD
ALK_37	2%	PDTC
ALK_37	69%	SD
ALK_39	-3%	SD
ALK_39	18%	SD
ALK_39	-9%	SD
ALK_40	-19%	SD
ALK_40	-44%	SD
ALK_40	19%	SD
ALK_43	2%	SD
ALK_43	61%	PDTC
ALK_43	-55%	SD
ALK_43	109%	SD
ALK_43	34%	SD
ALK_44	11%	SD
ALK_44	1%	PDTC
ALK_44	267%	PDTC
ALK_48	36%	SD
ALK_48	41%	SD
ALK_56	89%	SD
ALK_56	91%	PDTC
ALK_56	-67%	SD
ALK_56	-64%	SD
ALK_57	-40%	SD
ALK_59	9%	SD
ALK_60	-35%	SD
ALK_62	23%	SD
ALK_62	-45%	SD
ALK_62	-85%	SD
ALK_62	131%	SD
ALK_66	34%	SD
ALK_66	-44%	SD
ALK_66	58%	SD
ALK_101	34%	PDTC
ALK_101	144%	SD
ALK_101	-47%	SD
ALK_102	-18%	SD
ALK_104	92%	SD
ALK_104	-20%	SD
ALK_104	-19%	SD
ALK_105	27%	SD
ALK_105	40%	PDTC
ALK_105	5%	SD

SD: stable disease; PD: progressive disease; PDTC: progressive disease with therapy change

Supplementary table 5. ΔVAF_{mean} used for ROC analyses.

ALK_ID	ΔVAFmean	Clinical status
ALK_03	0.10%	PDTC
ALK_03	-0.10%	PDTC
ALK_03	0.00%	SD
ALK_03	0.00%	SD
ALK_03	0.22%	SD
ALK_03	0.11%	PDTC
ALK_03	0.06%	SD
ALK_03	-0.39%	SD
ALK_04	0.14%	SD
ALK_04	0.05%	PDTC
ALK_05	-0.05%	SD
ALK_06	0.00%	SD
ALK_06	0.00%	SD
ALK_06	0.34%	SD
ALK_06	-0.15%	SD
ALK_06	-0.08%	PDTC
ALK_07	0.54%	SD
ALK_07	-0.15%	PDTC
ALK_07	0.30%	SD
ALK_07	-0.13%	SD
ALK_08	0.21%	SD
ALK_08	-0.21%	SD
ALK_08	0.00%	SD
ALK_10	0.00%	SD
ALK_10	-0.07%	SD
ALK_12	-0.03%	SD
ALK_12	0.02%	SD
ALK_12	0.04%	SD
ALK_12	-0.10%	PDTC
ALK_12	0.21%	SD
ALK_12	-0.13%	SD
ALK_13	0.00%	SD
ALK_13	0.00%	SD
ALK_13	0.00%	SD
ALK_13	0.00%	SD
ALK_13	0.00%	SD
ALK_13	0.00%	SD
ALK_13	0.00%	SD
ALK_13	0.00%	SD
ALK_13	-0.73%	SD
ALK_13	2.38%	PDTC

ALK_13	-2.91%	SD
ALK_13	0.59%	SD
ALK_13	0.18%	SD
ALK_13	-0.10%	SD
ALK_13	-0.08%	SD
ALK_13	0.00%	SD
ALK_13	0.26%	SD
ALK_15	0.00%	SD
ALK_15	0.00%	SD
ALK_15	0.17%	PDTC
ALK_15	-0.17%	SD
ALK_15	0.00%	SD
ALK_15	0.00%	SD
ALK_15	0.00%	SD
ALK_16	0.00%	PDTC
ALK_16	0.00%	SD
ALK_18	0.51%	PDTC
ALK_18	0.00%	SD
ALK_18	0.79%	PDTC
ALK_18	2.52%	PDTC
ALK_18	-0.85%	SD
ALK_18	0.13%	SD
ALK_18	1.87%	SD
ALK_20	-0.14%	PDTC
ALK_20	0.29%	SD
ALK_20	-0.45%	SD
ALK_21	0.00%	SD
ALK_21	0.11%	PDTC
ALK_21	0.17%	SD
ALK_21	0.26%	PDTC
ALK_23	0.00%	SD
ALK_23	0.00%	SD
ALK_23	0.00%	SD
ALK_23	0.14%	SD
ALK_23	-0.14%	SD
ALK_23	0.00%	SD
ALK_24	0.00%	SD
ALK_24	0.04%	SD
ALK_24	-0.04%	SD
ALK_24	0.00%	SD
ALK_24	0.05%	SD
ALK_24	-0.05%	SD
ALK_24	0.00%	SD
ALK_24	0.00%	SD

ALK_25	-0.26%	SD
ALK_25	0.52%	SD
ALK_25	-0.04%	PDTC
ALK_25	0.57%	SD
ALK_25	0.11%	SD
ALK_26	0.00%	SD
ALK_26	0.02%	SD
ALK_26	0.10%	SD
ALK_26	-0.12%	SD
ALK_26	0.13%	SD
ALK_27	0.61%	PDTC
ALK_27	-0.55%	SD
ALK_27	-0.06%	SD
ALK_27	0.00%	SD
ALK_27	0.00%	SD
ALK_28	0.00%	SD
ALK_28	0.11%	SD
ALK_28	1.83%	PDTC
ALK_28	2.63%	PDTC
ALK_28	-0.67%	PDTC
ALK_28	0.18%	PDTC
ALK_29	0.00%	SD
ALK_29	0.00%	SD
ALK_30	0.00%	SD
ALK_30	0.00%	SD
ALK_30	0.11%	SD
ALK_30	-0.11%	PDTC
ALK_30	0.00%	SD
ALK_30	0.00%	SD
ALK_30	0.00%	SD
ALK_30	0.16%	SD
ALK_30	0.28%	PDTC
ALK_30	4.77%	PDTC
ALK_30	0.69%	PDTC
ALK_31	-0.16%	SD
ALK_32	0.00%	SD
ALK_32	0.00%	PDTC
ALK_32	0.04%	SD
ALK_32	-0.04%	SD
ALK_34	-0.19%	SD
ALK_34	0.43%	SD
ALK_34	-0.21%	SD
ALK_34	-0.22%	SD
ALK_35	0.00%	SD

ALK_35	0.00%	PDTC
ALK_35	0.00%	SD
ALK_39	0.00%	SD
ALK_39	0.00%	SD
ALK_39	0.08%	SD
ALK_40	0.05%	SD
ALK_40	0.21%	SD
ALK_40	0.28%	SD
ALK_43	0.00%	SD
ALK_43	0.00%	PDTC
ALK_43	0.00%	SD
ALK_43	0.20%	SD
ALK_43	0.00%	SD
ALK_44	0.00%	SD
ALK_44	0.09%	PDTC
ALK_44	0.27%	PDTC
ALK_48	0.00%	SD
ALK_48	0.00%	SD
ALK_56	0.13%	SD
ALK_56	2.61%	PDTC
ALK_56	-2.74%	SD
ALK_56	0.10%	SD
ALK_62	0.03%	SD
ALK_62	-0.03%	SD
ALK_62	-2.40%	SD
ALK_101	-0.14%	PDTC
ALK_101	2.60%	SD
ALK_101	-2.11%	SD
ALK_102	0.00%	SD
ALK_104	-0.11%	SD
ALK_104	0.36%	SD
ALK_104	-0.29%	SD
ALK_104	0.00%	SD

SD: stable disease; PD: progressive disease; PDTC: progressive disease with therapy change
