# Supplementary material for: Early identification of disease progression in ALK-rearranged lung cancer using circulating tumor DNA analysis
Source: NPJ Precis Oncol. 2021 Dec 7;5:100. doi: 10.1038/s41698-021-00239-3 (PMC8651695; doi:10.1038/s41698-021-00239-3)
Supplement: Supplementary file 2 — Reporting Summary [file 41698_2021_239_MOESM2_ESM.pdf]

nature research

Corresponding author(s): Holger Sültmann

Last updated by author(s): Sep 16, 2021

Reporting Summary

Nature Research wishes to improve the reproducibility of the work that we publish. This form provides structure for consistency and transparency in reporting. For further information on Nature Research policies, see our Editorial Policies and the Editorial Policy Checklist.

Statistics

For all statistical analyses, confirm that the following items are present in the figure legend, table legend, main text, or Methods section.

n/a	Confirmed	
☐	☒	The exact sample size (*n*) for each experimental group/condition, given as a discrete number and unit of measurement
☐	☒	A statement on whether measurements were taken from distinct samples or whether the same sample was measured repeatedly
☐	☒	The statistical test(s) used AND whether they are one- or two-sided<br>*Only common tests should be described solely by name; describe more complex techniques in the Methods section.*
☒	☐	A description of all covariates tested
☒	☐	A description of any assumptions or corrections, such as tests of normality and adjustment for multiple comparisons
☐	☒	A full description of the statistical parameters including central tendency (e.g. means) or other basic estimates (e.g. regression coefficient) AND variation (e.g. standard deviation) or associated estimates of uncertainty (e.g. confidence intervals)
☐	☒	For null hypothesis testing, the test statistic (e.g. *F*, *t*, *r*) with confidence intervals, effect sizes, degrees of freedom and *P* value noted<br>*Give P values as exact values whenever suitable.*
☒	☐	For Bayesian analysis, information on the choice of priors and Markov chain Monte Carlo settings
☒	☐	For hierarchical and complex designs, identification of the appropriate level for tests and full reporting of outcomes
☒	☐	Estimates of effect sizes (e.g. Cohen's *d*, Pearson's *r*), indicating how they were calculated

Our web collection on statistics for biologists contains articles on many of the points above.

Software and code

Policy information about availability of computer code

Data collection	Targeted panel sequencing data was generated using the Illumina NextSeq 550 platform with the High-Output Kit V2 (2 x 150 bp). Shallow whole genome sequencing data was generated using the Illumina HiSeq 4000 platform (2 x 100 bp).
Data analysis	One Touch Pipeline<br>AVENIO ctDNA analysis software (Roche Diagnostics, version 2.0.0)<br>R (version 3.3.1)<br>ichorCNA in R (version 3.3.1)<br>CNAclinic in R (version 3.5.2)<br>GraphPad Prism 9

For manuscripts utilizing custom algorithms or software that are central to the research but not yet described in published literature, software must be made available to editors and reviewers. We strongly encourage code deposition in a community repository (e.g. GitHub). See the Nature Research guidelines for submitting code & software for further information.

Data

Policy information about availability of data

All manuscripts must include a data availability statement. This statement should provide the following information, where applicable:

- Accession codes, unique identifiers, or web links for publicly available datasets
- A list of figures that have associated raw data
- A description of any restrictions on data availability

All sequencing data supporting the findings in this study are deposited in the European Genome-phenome Archive (EGA) under accession number EGAS00001005327.

Field-specific reporting

Please select the one below that is the best fit for your research. If you are not sure, read the appropriate sections before making your selection.

☒ Life sciences ☐ Behavioural & social sciences ☐ Ecological, evolutionary & environmental sciences

For a reference copy of the document with all sections, see nature.com/documents/nr-reporting-summary-flat.pdf

Life sciences study design

All studies must disclose on these points even when the disclosure is negative.

Sample size	No sample size estimation was performed as this is a study demonstrating method feasibility rather than discriminatory power of this analysis.
Data exclusions	Data from 41 patients corresponding to 69 plasma samples were excluded from the analysis, as detailed in the manuscript.
Replication	Not applicable.
Randomization	Not applicable.
Blinding	No blinding has been performed.

Reporting for specific materials, systems and methods

We require information from authors about some types of materials, experimental systems and methods used in many studies. Here, indicate whether each material, system or method listed is relevant to your study. If you are not sure if a list item applies to your research, read the appropriate section before selecting a response.

Materials & experimental systems

n/a	Involved in the study	
☒	☐	Antibodies
☒	☐	Eukaryotic cell lines
☒	☐	Palaeontology and archaeology
☒	☐	Animals and other organisms
☐	☒	Human research participants
☒	☐	Clinical data
☒	☐	Dual use research of concern

Methods

n/a	Involved in the study	
☒	☐	ChIP-seq
☒	☐	Flow cytometry
☒	☐	MRI-based neuroimaging

Human research participants

Policy information about studies involving human research participants

Population characteristics	ALK+ patients with metastatic NSCLC under TKI therapy were considered for the study. The median age of the study population (n=43) was 57 years (range 39-80), with almost equal distribution of males and females. Seventy eight percent of patients were never/light smokers (<10 pack-years). Newly diagnosed cases were screened for the presence of an ALK alteration by fluorescence in situ hybridization (FISH, ZytoLight SPEC ALK probe, ZytoVision GmbH, Bremerhaven, Germany) and reverse-transcription polymerase chain reaction until 2015, or by immunohistochemistry (D5F3 clone, Roche, Mannheim, Germany) and RNA-based next-generation sequencing (NGS, ThermoFisher Lung Cancer Fusion Panel, Waltham, MA, USA).
Recruitment	The study was approved by the ethics committees of Heidelberg University (S-270/2001, S-296/2016) and Lübeck University (AZ 12-238). Written informed consent was obtained from all patients. The patients were recruited at Thoraxklinik Heidelberg and Lungenclinic Grosshansdorf, Germany.
Ethics oversight	Ethics committees of Heidelberg University and Lübeck University.

Note that full information on the approval of the study protocol must also be provided in the manuscript.
